# Comparative analysis of surgical interventions for osteonecrosis of the femoral head: a network meta-analysis of randomized controlled trials

**DOI:** 10.1186/s13018-023-04463-4

**Published:** 2023-12-14

**Authors:** Liyou Hu, Xiaolei Deng, Bo Wei, Jian Wang, Decai Hou

**Affiliations:** 1 Liaoning University of Traditional Chinese Medicine, Shenyang, 110032 China; 2 Affiliated Hospital of Liaoning University of Traditional Chinese Medicine, Shenyang, 110032 China

**Keywords:** Femoral head osteonecrosis, Bone graft, Network meta-analysis, Surgical intervention, Clinical randomized controlled trials

## Abstract

**Background:**

Despite several surgical options, there remains no consensus regarding the optimal approach for osteonecrosis of the femoral head (ONFH), a prevalent and refractory disease. To determine the most suitable treatment modality, we compared randomized controlled trials (RCTs) that evaluated multiple surgical treatments for ONFH using a Bayesian network meta-analysis (NMA).

**Methods:**

The outcomes of 11 different surgical treatments were assessed using NMA comparisons of the rate of progression of femoral head necrosis, the rate of conversion to total hip arthroplasty, and improvement of the Harris hip score (HHS). A random effects model was used to analyze the odds ratio (OR) or mean difference, and risk of bias was assessed using the Cochrane risk of bias assessment tool for randomized trials. The confidence of the results was assessed using the confidence in network meta-analysis tool.

**Results:**

A total of 18 RCTs were included in the meta-analysis. Compared with core decompression (CD), the forest plot showed that autologous bone grafting (ABG), free fibula grafting (FFG), vascularized bone grafting (VBG), autologous bone grafting combined with bone marrow aspirate concentrate (ABG + BMAC), and biomaterial grafting combined with vascularized bone grafting (BMG + VBG) delayed ONFH progression. Among them, ABG + BMAC showed the most promising results (OR 0.019; 95% confidence interval [CI] 0.0012–0.25). However, upon comparing CD with different surgical modalities, no significant differences were found in preventing total hip arthroplasty. Furthermore, we cannot draw conclusions regarding the HHS due to attribution and high heterogeneity across the studies.

**Conclusion:**

Overall, ABG, VBG, FFG, ABG + BMAC, and BMG + VBG showed significant results in preventing ONFH progression compared with that shown by CD. Based on the surface under the cumulative ranking, ABG + BMAC was the most effective. Moreover, all treatments involving bone grafting were found to be effective, possibly indicating the necessity of its use in the treatment of ONFH.

**Supplementary Information:**

The online version contains supplementary material available at 10.1186/s13018-023-04463-4.

## Background

Osteonecrosis of the femoral head (ONFH), also known as avascular or aseptic necrosis of the femoral head, is a commonly encountered refractory disease in the field of orthopedics. Its prevalence is increasing annually [1], and approximately 12% of total hip arthroplasty (THA) procedures are performed to treat ONFH [2]. ONFH is a progressive disease typically caused by insufficient blood supply to the femoral head, resulting in increased pressure that may lead to femoral head collapse. Furthermore, secondary arthritis often develops at the site of collapse [3].

Several surgical interventions have had good results in the treatment of ONFH. Such methods include autologous bone grafting (ABG), bone marrow aspirate concentrate (BMAC), biomaterial grafting (BMG), vascularized bone grafting (VBG), autologous bone grafting and bone marrow aspirate concentrate (ABG + BMAC), biomaterial grafting and vascularized bone grafting (BMG + VBG), bone marrow aspirate concentrate and biomaterial grafting (BMAC + BMG), core decompression (CD), free fibular grafting (FFG), osteoblastic cells (OB), and platelet-rich plasma (PRP) [4–25].

BMG using β-tricalcium phosphate bioceramic materials shows superior results in osteogenesis, osteoinduction, osteoconduction, biodegradability, and cellular compatibility. The implantation of bioceramic materials additionally provides mechanical support, thereby avoiding femoral head collapse [26, 27]. ABG, VBG, and FFG have been shown to provide mechanical support to a certain extent, pose low risk of immune rejection, and be associated with low infection rates. ABG, such as with autogenous ilium, also includes the bone marrow, which can repair femoral head necrosis to a certain extent. However, this method still has certain restrictions due to its limited resources [9, 28]. BMAC, on the other hand, makes use of stem cells and endothelial progenitor cells, which have numerous biological properties, as well as non-cellular components, including cytokines and growth factors, which may work together to promote femoral head repair [8]. Another method, the use of OB cells, has been shown to be able to be differentiated by stem cells in BMAC, promote osteogenesis, and enhance the treatment of femoral head necrosis [8, 10]. Lastly, PRP has been shown to increase the concentration and release of various growth and differentiation factors at the site of injury, thus supplementing the healing process [18]. Despite the promising results of these different methods, the best surgical modality for ONFH has not yet been determined. Therefore, we conducted a network meta-analysis (NMA) to determine the best treatment option.

Bayesian NMA, also known as a multiple treatment comparison meta-analysis, is performed to simultaneously analyze direct and indirect evidence from a group of studies, expand the scope of traditional conventional pairwise analyses, estimate the relative effectiveness of all interventions, and rank such interventions [29]. In the present study, Bayesian NMA was performed to evaluate the efficacy of different surgical therapies based on ONFH progression, conversion to THA, and improvement of the Harris hip score (HHS) to determine the ideal surgical treatment for ONFH.

## Methods

This systematic review and NMA are reported in accordance with the preferred reporting items for systematic reviews and meta-analyses (PRISMA) statement [30]. This study was registered on the PROSPERO international prospective register of systematic reviews (CRD42023442015).

### Search strategy

The Cochrane Central Register of Controlled Trials of the Cochrane Library, Web of Science, PubMed, and EMBASE databases were systematically searched from their inception to June 20, 2023. The search strategy, which was developed in collaboration with a competent academic librarian, was based on established MESH and EMBASE search terms. The following keywords were used: femoral head, osteonecrosis, and randomized controlled trials (RCTs). The publication language did not limit the results. The complete search strategy is shown in (Additional file 1: Search Terms).

### Study inclusion and exclusion criteria

The inclusion criteria for the studies were as follows: RCT; all patients ≥ 18 years of age; non-traumatic ONFH; at least one group of patients received surgical treatment; included at least one case of ONFH progression, conversion to THA, and HHS improvement; and follow-up period ≥ 6 months. The exclusion criteria for the studies were as follows: studies with traumatic ONFH diagnoses; studies other than RCTs; and reviews and protocols.

### Study selection

The literature search was performed by two independent authors (J.W. and B.W.). The retrieved articles were imported into Endnote 20 to remove duplicate studies, and the remaining titles and abstracts were assessed to determine their eligibility. Following this, the full texts were reviewed to determine their eligibility. Disagreements during each step of this process were discussed with a third senior professor (D.H.) to reach a consensus.

### Data extraction and risk of bias assessment

The two authors (J.W. and B.W.) independently extracted the following data from each RCT: first author name, year of publication, host country, follow-up duration, ONFH staging, surgical intervention, number of hips included in the study, and mean patient age at baseline. Then, the following three outcomes were separately analyzed: ONFH progression, conversion to THA, and HHS improvement.

We used the confidence in network meta-analysis (CINeMA) tool to evaluate the credibility of the results [31]. Risk of bias assessment was conducted independently by two other authors (L.H. and X.D.) using the Cochrane risk of bias assessment tool for RCTs [32]. The following seven domains were examined: random sequence generation, allocation concealment, blinding of participants and personnel, blinding of outcome assessment, incomplete outcome data, selective reporting, and other sources of bias. Each domain was then determined as low, high, or unclear risk. Funnel plots were used to assess publication bias. Any differences in the assessments were resolved via discussions with a third senior professor (D.H.).

### Data synthesis and statistical analysis

Data were analyzed using the gemtc, coda, and rjags data packages in R software (version 5.35; Lucent Technologies, Paris, France). Odds ratios (ORs) and 95% confidence intervals (CIs) were used to compare the rates of ONFH progression and conversion to THA, whereas mean difference (MD) and 95% CIs were used to compare HHS improvement. Random effects models were used to compare the treatment outcomes [33, 34]. The interventions were ranked using the surface under the cumulative ranking (SUCRA), ranging from 0 to 1 with higher scores indicating better outcomes, and the most frequent analog of the SUCRA curve was determined [34–36].

We used the *I*^2^ statistic (*I*^2^ > 50%) to determine heterogeneity. A node-splitting analysis was used to assess inconsistency using specific comparisons based on direct and indirect evidence. For all analyses, *P* > 0.05 was considered insignificant [37].

## Results

### Search results

**Fig. 1:**
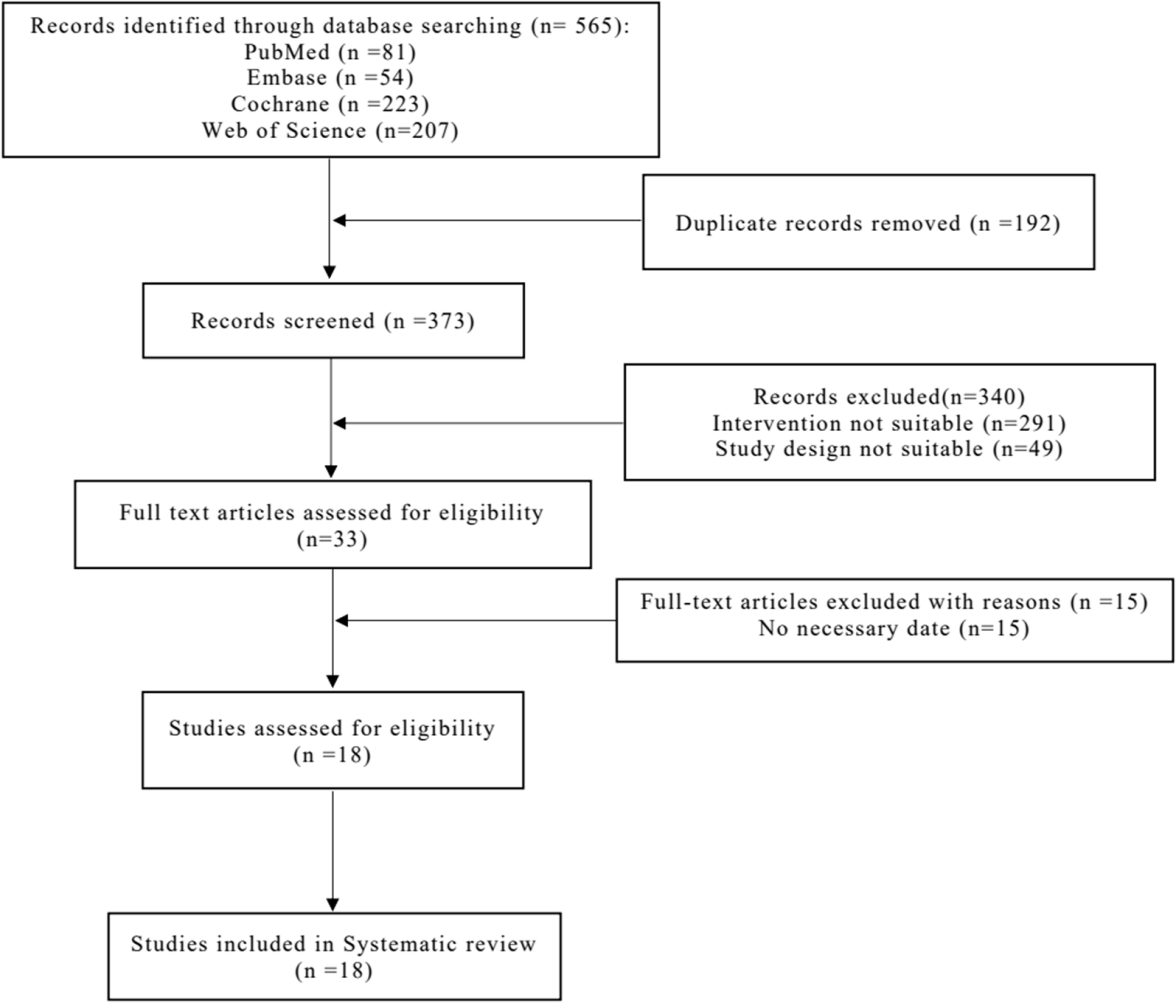
Flow chart of the study selection

Among the 565 articles yielded from the systematic search, 373 were found to be unique. After reviewing their titles and abstracts, only 33 articles underwent full-text review. Finally, 18 RCTs including 1107 hips met the NMA inclusion criteria (Fig. 1).

### Risk of bias and heterogeneity

**Fig. 2:**
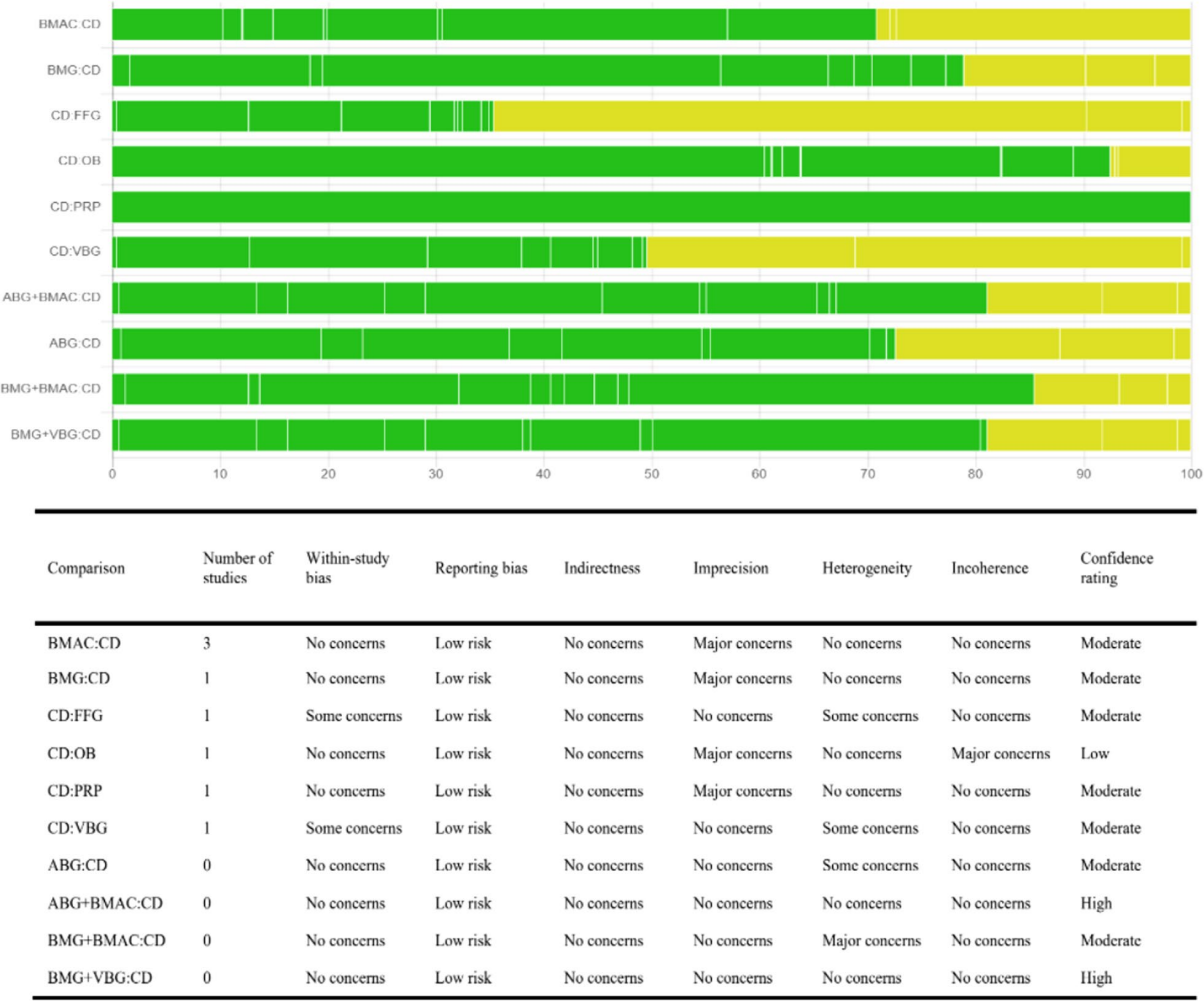
Results of the CINeMA assessment

**Fig. 3:**
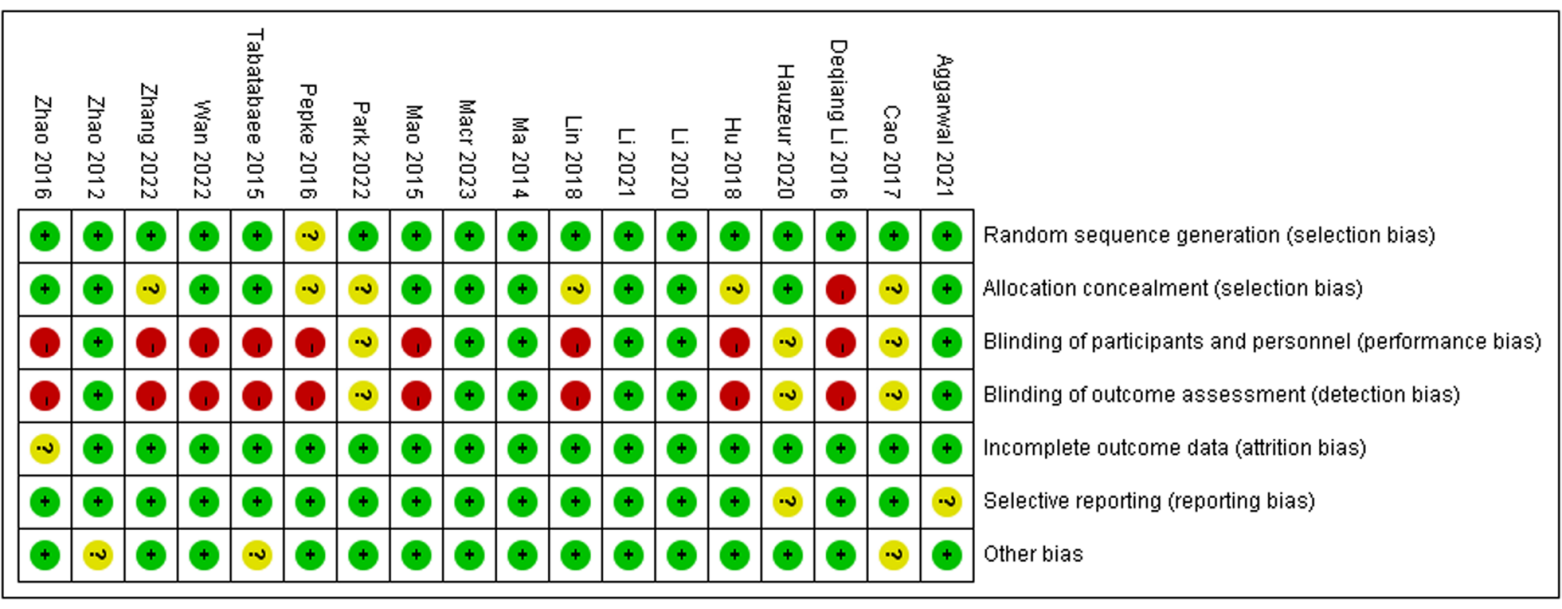
Details of the risks of bias of the included trials

**Fig. 4:**
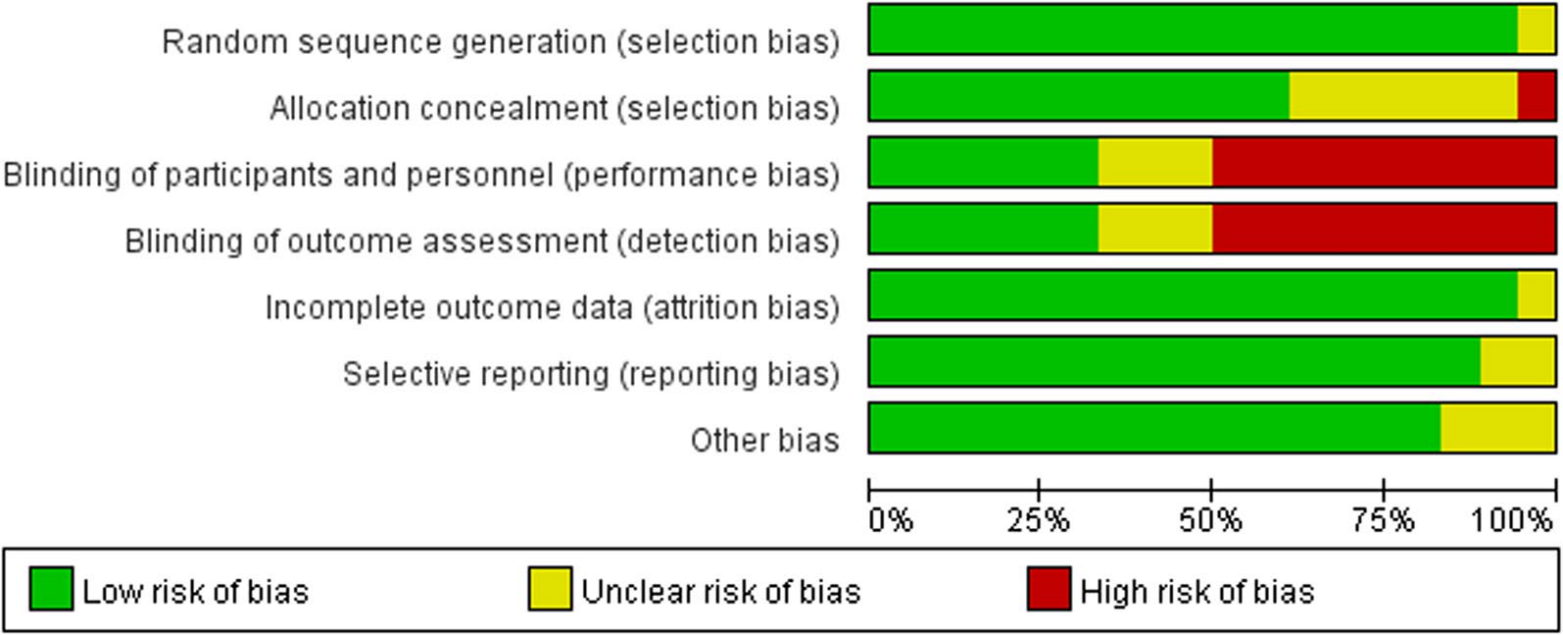
Summary of the risks of bias of the included trials

**Fig. 5:**
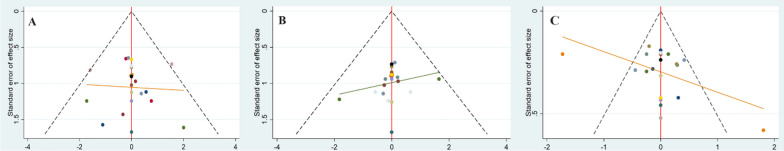
Possibility of publication bias as assessed by the comparison-adjusted funnel plot. **A** ONFH progression, **B** conversion to THA, **C** the improvement of HHS

Using the CINeMA tool, an overall moderate credibility was observed (Fig. 2). Based on the Cochrane Bias Risk Assessment Tool, the risk of bias in the generation of random sequences was low and in allocation concealment was moderate. In some studies biases were identified in participant and personnel blinding and outcome assessment blinding. The risks of bias in incomplete outcome data, selective reporting, and other sources of bias were low. The overall quality of the 18 included studies was moderate, and the results are shown in Figs. 3 and 4. The funnel plot indicated no significant publication bias (Fig. 5). Heterogeneity analysis showed low heterogeneity in ONFH progression and conversion to THA across the different treatment methods, although high heterogeneity was noted in HHS improvement (Additional file 2: Results of heterogeneity according to pairwise meta-analysis). Furthermore, node-splitting analysis showed no significant inconsistency between direct and indirect evidence (Additional file 3: The results of inconsistency assessment).

### Study characteristics

The characteristics of the 18 RCTs included in the NMA are presented in Table 1. A total of 1107 hips were included, and the follow-up period ranged from 12 to 36 months. All studies included patients ≥ 18 years of age. Regarding country, 12 studies were conducted in China, two were conducted in Belgium, and one each was conducted in Korea, Germany, India, and Iran. Regarding outcomes, 18 reported ONFH progression, 16 reported conversion to THA, and 13 reported HHS improvement.

**Table 1:** Characteristics of studies included in the meta-analysis

References	Year	Country	Design	Treatment	Mean age(years)	Case(hip)	Inclusion criteria	Follow-up (months)	Outcomes
Jayankura et al. [10]	2023	Belgium	RCT	OB	46	23	ARCO I–II	24	Progress, THA
				CD	45	26			
Wan et al. [9]	2022	China	RCT	FFG	29.8	45	ARCO II	36	HHS, progress, THA
				VBG	28.8	46			
				ABG	30.5	45			
				BMG	30.5	46			
Zhang et al. [19]	2022	China	RCT	FFG	34.3	29	ARCO II	24	HHS, progress, THA
				VBG	34.4	30			
Park et al. [12]	2022	Korea	RCT	BMG	49.3	10	Ficat I–II	36	HHS, progress, THA
				CD	55.6	10			
Aggarwal et al. [18]	2021	India	RCT	PRP	35.2	25	ARCO I–II	12	Progress, THA
				CD	38.2	28			
Li et al. [7]	2021	China	RCT	BMAC	35.4	22	Ficat I–IV	24	HHS, progress, THA
				BMG	39.4	29			
Li et al. [22]	2020	China	RCT	ABG	38.2	20	Ficat II–III	24	Progress, THA
				BMAC + ABG	34.1	21			
Hauzeur et al. [8]	2020	Belgium	RCT	BMAC	50	26	ARCO I–II	36	Progress, THA
				OB	51	27			
Hu et al. [4]	2018	China	RCT	CD	40.3	65	ARCO I–II	24	HHS, progress
				FFG	40.8	65			
Lin et al. [17]	2018	China	RCT	BMG	31.5	16	ARCO II–III	36	HHS, progress, THA
				ABG	32.6	16			
Cao et al. [20]	2017	China	RCT	VBG	31	27	ARCO I–III	36	HHS, progress, THA
				CD		27			
Li et al. [21]	2016	China	RCT	ABG	36.5	23	Ficat I–II	24	HHS, progress, THA
				VBG		24			
Zhao et al. [23]	2016	China	RCT	ABG	33	25	ARCO II–III	12	HHS, progress
				BMG + VBG	30	23			
Pepke et al. [11]	2016	Germany	RCT	BMAC	44.5	14	ARCO II	24	HHS, progress, THA
				CD	44.3	11			
Tabatabaee et al. [24]	2015	Iran	RCT	BMAC	29.1	14	ARCO I–III	24	Progress, THA
				CD		14			
Mao et al. [13]	2015	China	RCT	BMAC: + BMG	34.6	48	ARCO I–III	36	HHS, progress, THA
				BMG	36.1	41			
Ma et al. [5]	2014	China	RCT	ABG	34.8	24	Ficat I–III	24	Progress, THA
				BMAC + ABG	35.6	25			
Zhao et al. [14]	2012	China	RCT	CD	33.8	44	ARCO I–II	24	HHS, progress, THA
				BMAC	32.7	53			

*ABG* autologous iliac bone grafting, *ABG* + *BMAC* autologous bone grafting and bone marrow aspirate concentrate, *BMAC* bone marrow aspirate concentration, *BMG* biomaterials grafting, *BMG* + *BMAC* biomaterials grafting and bone marrow aspirate concentration, *CI* confidence interval, *CD* core decompression, *FFG* free fibular graft, *HHS* Harris hip score, *MD* mean difference, *NMA* network meta-analysis, *OR* odds ratio, *ONFH* osteonecrosis of the femoral head, *OB* osteoblastic cells, *PRP* platelet-rich plasma, *RCT* randomized controlled trial, *THA* total hip arthroplasty, *VBG* vascularized bone grafting, *VBG* + *BMG* vascularized bone grafting combined with biomaterial

### Progression of ONFH

**Fig. 6:**
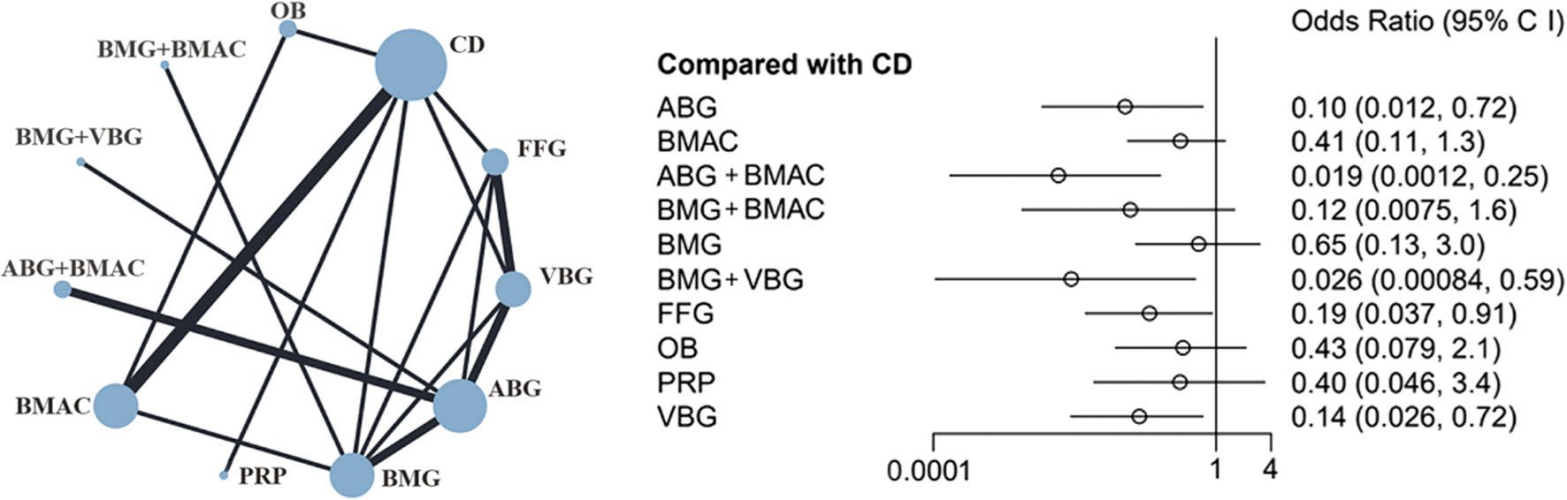
Results of network comparisons for progression of ONFH

The results of the joint intervention network and forest plot based on 11 surgical interventions are shown in Fig. 6. ABG + BMAC was identified as most effective for preventing ONFH progression (OR 0.019; 95% CI 0.0012–0.25; SUCRA = 0.926), followed by BMG + VBG (OR 0.026; 95% CI 0.0008–0.59; SUCRA = 0.865), ABG (OR 0.10; 95% CI 0.012–0.72; SUCRA = 0.668), VBG (OR 0.14; 95% CI 0.026–0.72; SUCRA = 0.603), and FFG (OR 0.19; 95% CI 0.037–0.91; SUCRA = 0.535). The SUCRA values of ONFH progression are shown in Table 2. On comparing the progression of each group, differences among the BMG + BMAC, BMAC, OB cell, and PRP treatment groups were insignificant. A net league table of the treatment groups is shown in Table 3.

**Table 2:** SUCRA value of each group

Surgical intervention	ONFH progression	Conversion to THA	The improvement of HHS
ABG	0.669	0.236	0.322
BMAC	0.341	0.630	0.570
BMG	0.192	0.403	0.338
CD	0.075	0.335	0.071
FFG	0.535	0.213	0.611
OB	0.321	0.571	–
PRP	0.341	0.702	0.716
VBG	0.603	0.392	0.510
ABG + BMAC	0.926	0.725	–
BMG + BMAC	0.632	0.790	0.805
BMG + VBG	0.865	–	0.557

**Table 3:** Net league table of ONFH progression

ABG										
0.25 (0.03, 2.4)	BMAC									
5.28 (1.01, 30.92)	21.07 (1.27, 351.97)	ABG + BMAC								
0.88 (0.05, 14.61)	3.49 (0.2, 55)	0.16 (0.01, 4.22)	BMG + BMAC							
0.16 (0.03, 0.86)	0.64 (0.1, 3.36)	0.03 (0, 0.32)	0.18 (0.02, 1.56)	BMG						
3.79 (0.33, 60.21)	15.31 (0.54, 504.43)	0.72 (0.04, 17.51)	4.39 (0.11, 219.55)	24.16 (1.23, 654.04)	BMG + VBG					
0.1 (0.01, 0.73)	0.41 (0.11, 1.26)	0.02 (0.01, 0.25)	0.12 (0.01, 1.6)	0.65 (0.13, 2.99)	0.03 (0.01, 0.59)	CD				
0.53 (0.07, 3.92)	2.11 (0.29, 14.02)	0.1 (0.01, 1.34)	0.61 (0.03, 9.79)	3.31 (0.55, 20.56)	0.14 (0, 3.21)	5.28 (1.08, 27.7)	FFG			
0.23 (0.02, 2.92)	0.94 (0.17, 4.53)	0.04 (0, 0.91)	0.27 (0.01, 5.75)	1.48 (0.17, 13.02)	0.06 (0, 2.05)	2.35 (0.47, 12.46)	0.44 (0.05, 4.33)	OB		
0.25 (0.01, 4.65)	1.01 (0.08, 10.93)	0.05 (0, 1.35)	0.29 (0.01, 8.71)	1.59 (0.11, 22.49)	0.06 (0, 2.91)	2.5 (0.29, 22.07)	0.47 (0.03, 7.01)	1.07 (0.07, 15.61)	PRP	
0.69 (0.11, 4.27)	2.7 (0.38, 19.51)	0.13 (0.01, 1.52)	0.78 (0.05, 13.11)	4.29 (0.78, 26.45)	0.18 (0.01, 3.91)	6.78 (1.4, 40.08)	1.28 (0.29, 6.41)	2.88 (0.3, 30.21)	2.7 (0.19, 43.94)	VBG

### Conversion to THA

**Fig. 7:**
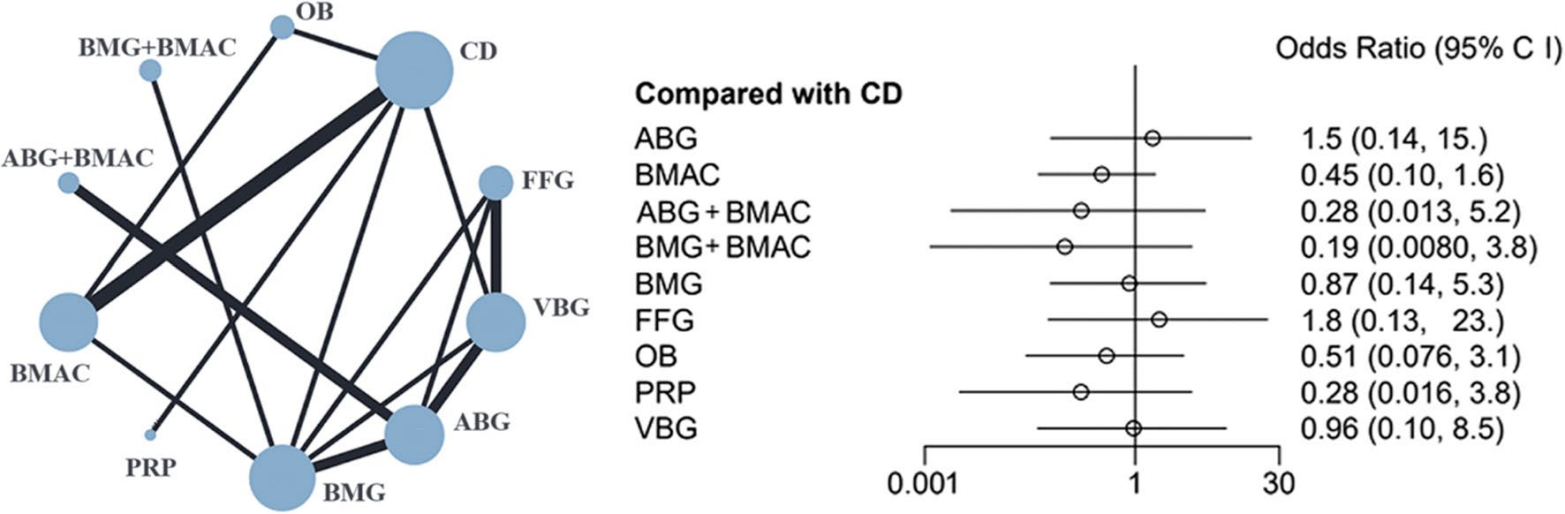
Results of network comparisons for conversion to THA

The results of the joint intervention network and forest plot formed by 10 surgical interventions are shown in Fig. 7. No significant differences in conversion to THA were detected among the treatment groups. The SUCRA values for conversion to THA are shown in Table 2, and a net league table of the treatment groups is shown in Table 4.

**Table 4:** Net league table of conversion to THA

ABG									
3.25 (0.29, 43.81)	BMAC								
5.29 (0.88, 35.47)	1.64 (0.07, 34.74)	ABG + BMAC							
7.95 (0.39, 182.74)	2.5 (0.1, 57.95)	1.49 (0.04, 55.57)	BMG + BMAC						
1.68 (0.29, 10.62)	0.53 (0.07, 3.43)	0.32 (0.02, 4.13)	0.21 (0.02, 2.42)	BMG					
1.5 (0.14, 15)	0.45 (0.1, 1.6)	0.28 (0.01, 5.2)	0.19 (0.01, 3.8)	0.87 (0.14, 5.3)	CD				
0.85 (0.1, 6.89)	0.26 (0.02, 3.65)	0.16 (0.01, 2.52)	0.11 (0, 2.74)	0.5 (0.06, 4.29)	0.57 (0.04, 7.55)	FFG			
2.92 (0.16, 54.13)	0.9 (0.13, 5.28)	0.54 (0.02, 16.92)	0.36 (0.01, 12.15)	1.7 (0.15, 20.91)	1.95 (0.32, 13.03)	3.44 (0.16, 76.95)	OB		
5.47 (0.16, 216.32)	1.65 (0.08, 35.81)	1.02 (0.02, 61.15)	0.68 (0.01, 42.59)	3.18 (0.13, 92.36)	3.62 (0.27, 61.82)	6.45 (0.16, 289.26)	1.87 (0.07, 53.38)	PRP	
1.55 (0.26, 9.2)	0.47 (0.04, 5.05)	0.29 (0.02, 3.64)	0.19 (0.01, 4.27)	0.9 (0.14, 6.1)	1.03 (0.12, 9.69)	1.8 (0.3, 11.61)	0.53 (0.03, 8.51)	0.28 (0.01, 8.91)	VBG

### Improvement of HHS

**Fig. 8:**
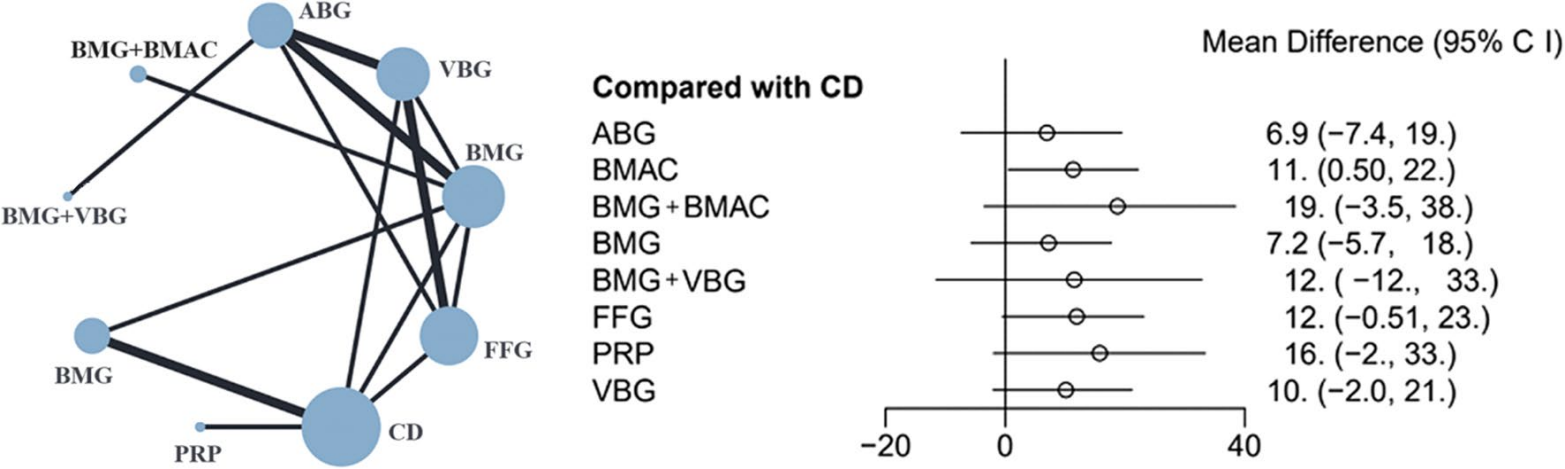
Results of network comparisons for improvement in HHS

The results of the joint intervention network and forest plot formed by 9 surgical interventions are shown in Fig. 8. The SUCRA values of the HHS are shown in Table 2, and a net league table of the treatment groups is shown in Table 5. Although the forest plot showed statistical significance with BMAC (MD: 11; 95% CI 0.5–22) than with CD, the heterogeneity analysis indicated high heterogeneity within the BMAC group. Therefore, the HHS improvement was inconclusive.

**Table 5:** Net league table of HHS improvement

CD								
6.9 (− 7.4, 19 2)	ABG							
7.2 (− 5.7, 18)	0.37 (− 11.16, 10.93)	BMG						
10.09 (− 2, 21.08)	3.17 (− 7.34, 14.32)	2.83 (− 8.03, 15.12)	VBG					
12 (− 12, 33.01)	4.58 (− 13.32, 22.47)	4.23 (− 16.36, 25.64)	1.41 (− 19.77, 21.97)	BMG + VBG				
11 (0.5, 22)	4.46 (− 10.42, 20.82)	4.12 (− 8.04, 18.62)	1.26 (− 12.86, 16.37)	− 0.14 (− 23.09, 24.29)	BMAC			
12 (− 0.51, 23)	5.01 (− 7.36, 17.8)	4.63 (− 7, 17.68)	1.83 (− 9.03, 12.56)	0.45 (− 21.24, 22.56)	0.58 (− 14.95, 14.89)	FFG		
16 (− 2, 33)	8.88 (− 12.59, 32.15)	8.54 (− 11.49, 31.18)	5.7 (− 15, 27.59)	4.34 (− 23.44, 33.69)	4.47 (− 16.42, 25.49)	3.88 (− 16.93, 26.05)	PRP	
19 (− 3.5, 38)	11.85 (− 9.34, 32.04)	11.46 (− 5.96, 28.97)	8.68 (− 12.92, 28.77)	7.25 (− 20.48, 34.09)	7.37 (− 15.67, 28.15)	6.84 (− 15.15, 27.68)	2.95 (− 25.93, 29.17)	BMG + BMAC

## Discussion

In this study, the NMA included 18 RCTs, which analyzed the outcomes of 11 different surgical approaches for ONFH. Upon analyzing these results, we found that ABG + BMAC, BMG + VBG, ABG, VBG, and FFG prevented ONFH progression. Based on the SUCRA values, ABG + BMAC was the most effective treatment. In addition, all treatments involving bone grafting were effective in preventing ONFH progression, indicating the necessity of its use in ONFH treatment. To the best of our knowledge, this is the first NMA to systematically compare the efficacy of different hip-sparing procedures for ONFH.

Although CD is commonly used in clinical practice, its effectiveness in preventing femoral head collapse or conversion to THA is controversial [38]. Some scholars believe that although CD reduces the internal pressure of the femoral head, the area of necrosis lacks support. The femoral head would eventually collapse, and THA will ultimately become necessary [39]. Thus, supportive material at the area of necrosis may be a viable option to prevent this. Currently, grafting materials, including autogenous bone, biomaterials, and metallic materials, are commonly used. Among them, bone materials are most readily available and can be classified as autogenous iliac bone, autogenous fibula, and autogenous bone with free blood vessels [5, 7, 9, 20, 21]. The most common biomaterials are bioceramics [9, 12, 17], whereas the most common metallic materials are tantalum rods and biodegradable magnesium screws [23, 40]. Additionally, researchers have found that the use of implants with injections of bone marrow, stem cells, OB cells, and PRP showed better results [7, 8, 11, 18, 24]. For example, Jeyaraman et al. [41] found that stem cells can treat ONFH by relieving pain, significantly improving function, and delaying femoral head collapse. Similarly, the present study found that ABG + BMAC was the most effective of the 11 surgical interventions, possibly because it provides both mechanical support and regenerative capacity in the necrotic area of the femoral head. Moreover, there are other treatment options for ONFH, such as osteotomy, which seem to yield good treatment outcomes [42]. However, since they have not yet been investigated by RCTs, they were not included in the present study.

A traditional pairwise meta-analysis of surgical interventions for ONFH by Wang et al. [43] found that autologous bone marrow enrichment improved the efficacy of ONFH treatment, notably highlighting the superiority of VBG over CD. A study by Migliorini et al. [44] found that the combination of CD with bone marrow-derived cell therapies reduced pain and total hip replacement rates as compared to CD alone; however, comparisons with other treatment modalities were not done. Another study by Sadile et al. [38] found that joint-sparing treatment was superior to CD, although only prospective cohort studies were included. Traditional meta-analyses predominantly consist of pairwise comparisons, which have certain inherent limitations. One example is a previous NMA on bone transplantation which was limited due to its sole inclusion of bone transplantation-related surgeries and lack of comparisons with other treatments [45]. A study by Yu et al. [46] that compared different treatments for ONFH was also found to be limited due to the use of traditional NMA and the inclusion of a previous meta-analysis that assessed non-surgical treatment. Unlike these studies, the Bayesian NMA that we conducted allowed for direct and indirect comparisons between interventions using all available evidence in the network while retaining intra-trial randomization.

To the best of our knowledge, the current study is the first NMA to systematically summarize RCTs of different surgical treatments for ONFH to provide credible results. Furthermore, the study found that ABG + BMAC, BMG + VBG, ABG, VBG, and FFG prevented ONFH progression. However, there were no significant differences in conversion to THA among the surgical treatments. This is most likely because majority of the included studies were conducted in China, where patients have limitations regarding the use of artificial hip joints. Such patients may endure the pain of ONFH for up to 10 years before consenting to undergo THA. Moreover, since the follow-up time in the included articles ranged from 12 to 36 months, most of which were less than 36 months, it was apparent that Chinese patients were unlikely to undergo THA.

Despite the findings of this study, certain limitations were noted. First, only 18 relevant studies were included; thus, the scale of direct comparison was small. In addition, a sample size of 1107 hips reduced the power of the statistical analysis, which may have affected the reliability of the results. Second, the inclusion of patients with different stages of ONFH may have reduced the reliability of the results. Since different classifications may affect prognosis, the credibility of our conclusions would most likely be reduced. The included articles used various classification types, such as ARCO I–II, ARCO II, ARCO II–III, and Ficat I–IV. We also attempted to analyze the data of patients with the Ficat II/III classification; however, only few of the articles specifically diagnosed the patients as Ficat II/III. Furthermore, indirect and direct circular comparisons between interventions could not be performed for these patients. Lastly, age, sex, treatment time, and other factors may affect the prognosis of ONFH treatment [47]. Given that multiple factors can have affected the credibility of our results, further studies involving larger sample sizes and patients with the same ONFH classification are necessary.

## Conclusions

Overall, ABG, VBG, FFG, ABG + BMAC, and BMG + VBG showed significant results in preventing ONFH progression compared with that shown by CD. Based on the SUCRA, ABG + BMAC was the most effective treatment. Moreover, all treatments involving bone grafting were found to be effective, possibly indicating the necessity of its use in the treatment of ONFH.

### Supplementary Information

**Additional file 1: Appendix S1.** Search terms.

**Additional file 2: Supplementary Table 2.** Results of heterogeneity according to pairwise meta-analysis.

**Additional file 3.** The results of inconsistency assessment.

## Data Availability

The datasets used and/or analyzed during the current study are available from the corresponding author on reasonable request. The protocol for this meta-analysis was registered on PROSPERO (CRD42023442015) and is available at: https://www.crd.york.ac.uk/PROSPERO.

## Abbreviations

ABG: Autologous bone grafting
ABG + BMAC: Autologous bone grafting and bone marrow aspirate concentrate
BMAC: Bone marrow aspirate concentration
BMG: Biomaterials grafting
BMG + BMAC: Biomaterials grafting and bone marrow aspirate concentration
CI: Confidence interval
CD: Core decompression
FFG: Free fibular graft
HHS: Harris hip score
MD: Mean difference
NMA: Network meta-analysis
OR: Odds ratio
ONFH: Osteonecrosis of the femoral head
OB: Osteoblastic
PRP: Platelet-rich plasma
RCT: Randomized controlled trial
THA: Total hip arthroplasty
VBG: Vascularized bone grafting
VBG + BMG: Vascularized bone grafting combined with biomaterial grafting
