# Supplementary material for: Comparative analysis of surgical interventions for osteonecrosis of the femoral head: a network meta-analysis of randomized controlled trials
Source: J Orthop Surg Res. 2023 Dec 14;18:965. doi: 10.1186/s13018-023-04463-4 (PMC10722734; doi:10.1186/s13018-023-04463-4)
Supplement: Supplementary file 1 — Additional file 1: Appendix S1. Search terms. [file 13018_2023_4463_MOESM1_ESM.docx]

Additional file 1. Search Terms

**Pubmed (MEDLINE)**

((((((((((((((((("Osteonecrosis"[Mesh]) OR (Osteonecroses[Title/Abstract])) OR (Bone Necrosis[Title/Abstract])) OR (Bone Necroses[Title/Abstract])) OR (Necroses, Bone[Title/Abstract])) OR (Necrosis, Bone[Title/Abstract])) OR (Necrosis, Avascular, of Bone[Title/Abstract])) OR (Avascular Necrosis of Bone[Title/Abstract])) OR (Bone Avascular Necrosis[Title/Abstract])) OR (Kienbock Disease[Title/Abstract])) OR (Kienbock's Disease[Title/Abstract])) OR (Kienboeck Disease[Title/Abstract])) OR (Kienboeck's Disease[Title/Abstract])) OR (Kienboecks Disease[Title/Abstract])) OR (Necrosis, Aseptic, of Bone[Title/Abstract])) OR (Aseptic Necrosis of Bone[Title/Abstract])) OR (Bone Aseptic Necrosis[Title/Abstract])) AND (((((("Femur Head"[Mesh]) OR (Femur Heads[Title/Abstract])) OR (Head, Femur[Title/Abstract])) OR (Femoral Head[Title/Abstract])) OR (Femoral Heads[Title/Abstract])) OR (Head, Femoral[Title/Abstract])) AND (randomizedcontrolledtrial[Filter]) 81

**Cochrane Central Register of Controlled Trials (CENTRAL) in The Cochrane Library (Wiley)**

#1 MeSH descriptor: [Osteonecrosis] explode all trees 347

#2 (Osteonercroses): ti,ab,kw OR(Bone Necrosis):ti,ab,kw OR (Bone Avascular Necrosis):ti,ab,kw OR (Kienbock Disease):ti,ab,kw OR (Aseptic Necrosis of Bone):ti,ab,kw 1446

#3 MeSH descriptor: [Femur Head] explode all trees 129

#4 (Femur Heads): ti,ab,kw OR (Femoral Head):ti,ab,kw OR (Femoral Heads): ti,ab,kw 1409

#5 (random): ti,ab,kw 84752

#6 #1 OR #2 1655

#7 #3 OR #4 1423

#8 #5 AND #6 AND #7 223

**EMBASE (Ovid)**

#1 'bone necrosis'/exp 45868

#2 'osteonecroses': ti,ab 128

#3 'bone necrosis': ti,ab 1375

#4 'bone necroses': ti,ab 51

#5 'bone avascular necrosis': ti,ab 13

#6 'kienboeck disease': ti,ab 4

#7 'aseptic necrosis of bone': ti,ab 88

#8 #1 OR #2 OR #3 OR #4 OR #5 OR #6 OR #7 46333

#9 'femoral head'/exp 17737

#10 'femur heads': ti,ab 117

#11 'femoral head': ti,ab 21924

#12 'femoral heads': ti,ab 4441

#13 #9 OR #10 OR #11 OR #12 30040

#14 'random': ti,ab 426527

#15 #8 AND #13 AND #14 54

**Web of Science**

#1 (((((TS=(Osteonecroses*)) OR TS= (Bone Necrosis*)) OR TS= (Bone Necroses*)) OR TS= (Bone Avascular Necrosis*)) OR TS= (Kienbock Disease)) OR TS= (Aseptic Necrosis of Bone*) 30749

#2 ((TS=(Femur Heads*)) OR TS=(Femoral Head*)) OR TS=(Femoral Heads*) 21674

#3 TS=(random*) 2127044

#4 #1 AND #2 AND #3 207
