# Supplementary material for: Comparative analysis of surgical interventions for osteonecrosis of the femoral head: a network meta-analysis of randomized controlled trials
Source: J Orthop Surg Res. 2023 Dec 14;18:965. doi: 10.1186/s13018-023-04463-4 (PMC10722734; doi:10.1186/s13018-023-04463-4)
Supplement: Supplementary file 2 — Additional file 2: Supplementary Table 2. Results of heterogeneity according to pairwise meta-analysis. [file 13018_2023_4463_MOESM2_ESM.docx]

Additional file 2: Results of heterogeneity according to pairwise meta-analysis

| Comparison | Number of studies included | Conversion to THA | ONFH progression | HHS improvement |
| --- | --- | --- | --- | --- |
|  |  | I^2^ | I^2^ | I^2^ |
| BMG+VBG vs ABG | 2 | 0.0% | 11.1% | - |
| FFG vs ABG | 1 | - | - | - |
| VBG vs ABG | 2 | 0.0% | 0.0% | 95.2% |
| BMG vs BMAC | 1 | - | - | - |
| CD vs BMAC | 3 | 23.1% | 15% | 79.6% |
| BMG vs BMAC+BMG | 1 | - | - | - |
| CD vs BMG | 1 | - | - | - |
| FFG vs BMG | 1 | - | - | - |
| VBG vs BMG | 2 | 11.6% | 7.1% | - |
| FFG vs CD | 1 | - | - | - |
| OB vs CD | 1 | - | - | - |
| VBG vs CD | 1 | - | - | - |
| VBG vs FFG | 2 | 0.0% | 0.0% | 0.0% |
