# Supplementary material for: Comparative analysis of surgical interventions for osteonecrosis of the femoral head: a network meta-analysis of randomized controlled trials
Source: J Orthop Surg Res. 2023 Dec 14;18:965. doi: 10.1186/s13018-023-04463-4 (PMC10722734; doi:10.1186/s13018-023-04463-4)
Supplement: Supplementary file 3 — Additional file 3. The results of inconsistency assessment. [file 13018_2023_4463_MOESM3_ESM.docx]

Additional file 3A: The results of inconsistency assessment of ONFH progression by node-splitting analysis.

| **Name** | **Direct Effect** | **Indirect Effect** | **Overall** | **P Value** | **Number of studies included** |
| --- | --- | --- | --- | --- | --- |
| BMG vs ABG | 5.2(0.67,48) | 22(0.41,150) | 6.2(1.2,39) | 0.51 | 2 |
| FFG vs ABG | 2.5(0.1,200) | 1.4(0.11,22) | 1.8(0.25,15) | 0.78 | 1 |
| VBG vs ABG | 1.4(0.14,14) | 1.8(0.25,15) | 1.4(0.24,8.8) | 0.9 | 2 |
| BMG vs BMAC | 6.0(0.39,250) | 0.75(0.08,7.1) | 1.6(0.31,9.7) | 0.23 | 1 |
| CD vs BMAC | 3.5(0.83,16) | 1.2(0.17,15) | 2.5(0.80,9.2) | 0.41 | 3 |
| OB vs BMAC | 1.2(0.07,12) | 6.2(0.88,47) | 1.1(0.22,5.7) | 0.06 | 1 |
| CD vs BMG | 1.6(0.11,24) | 1.6(0.19,13) | 1.5(0.34,7.4) | 0.98 | 1 |
| FFG vs BMG | 1.0(0.05,19) | 0.12(0.01,1.3) | 0.29(0.05,1.7) | 0.24 | 1 |
| VBG vs BMG | 0.40(0.01,9.3) | 0.12(0.01,1.2) | 0.23(0.04,1.2) | 0.56 | 2 |
| FFG vs CD | 0.16(0.01,1.7) | 0.23(0.02,2.8) | 0.19(0.04,0.94) | 0.81 | 1 |
| OB vs CD | 1.5(0.28,1.6) | 0.07(0.01,0.53) | 0.43(0.08,2.1) | 0.07 | 1 |
| VBG vs CD | 0.05(0.01,0.79) | 0.25(0.03,2.1) | 0.15(0.03,0.72) | 0.36 | 1 |
| VBG vs FFG | 0.87(0.12,5.5) | 00.68(0.02,20) | 0.78(0.16,3.5) | 0.89 | 2 |

Additional file 3B: The results of inconsistency assessment of conversion to THA by node-splitting analysis.

| **Name** | **Direct Effect** | **Indirect Effect** | **Overall** | **P Value** | **Number of studies included** |
| --- | --- | --- | --- | --- | --- |
| BMG vs ABG | 0.41(0.048,3.1) | 5.1(0.034,120) | 0.58(0.093,3.5) | 0.34 | 2 |
| FFG vs ABG | 1.7(0.09,36) | 1.1(0.025,48) | 1.2(0.14,9.7) | 0.84 | 1 |
| VBG vs ABG | 1.2(0.18,9.2) | 0.027(0.001,1.4) | 0.65(0.11,3.7) | 0.08 | 2 |
| BMG vs BMAC | 6.1(0.33,280) | 0.83(0.059,13) | 1.9(0.29,15) | 0.30 | 1 |
| CD vs BMAC | 3.2(0.67,19) | 0.98(0.095,15) | 2.2(0.62,9.9) | 0.41 | 3 |
| OB vs BMAC | 0.3(0.7,2.2) | 7.6(0.73,97) | 1.1(0.19,7.7) | 0.06 | 1 |
| CD vs BMG | 1.6(0.094,26) | 0.87(0064,12) | 1.1(0.18,7.4) | 0.74 | 1 |
| FFG vs BMG | 1.0(0.062,17) | 6.8(0.13,380) | 2(0.23,18) | 0.41 | 1 |
| VBG vs BMG | 0.60(0.03,11) | 2.4(0.15,38) | 1.1(0.16,7.4) | 0.46 | 2 |
| OB vs CD | 2.1(0.27,17) | 0.4(0.04,0.82) | 0.51(0.075,3.1) | 0.07 | 1 |
| VBG vs CD | 0.39 (0.009,0.11) | 1.9(0.09 ,42) | 0.95(0.097,8.7) | 0.46 | 1 |

Additional file 3C: The results of inconsistency assessment of Harris Hip Score improvement by node-splitting analysis.

| **Name** | **Direct Effect** | **Indirect Effect** | **Overall** | **P Value** | **Number of studies included** |
| --- | --- | --- | --- | --- | --- |
| BMG vs ABG | 4.8(-1.8,11) | -5(-9.1,2) | 0.38(-4,11) | 0.06 | 2 |
| FFG vs ABG | 2(-19,23) | 9(-13,31) | 5(-7.3,18) | 0.58 | 1 |
| VBG vs ABG | -0.6(-12,11) | 18(-4.3,43) | 3.2(-7.3,14) | 0.11 | 2 |
| BMG vs BMAC | -13(-30,4.5) | 4.1(-14,18) | -4.1(-19,8) | 0.16 | 1 |
| CD vs BMAC | -8.5(-19,2.4) | -25(-44,-1.7) | -11(-22,-0.45) | 0.16 | 2 |
| CD vs BMG | 4.7(-16,25) | -12(-24,2.5) | -7.2(-18,5.6) | 0.18 | 1 |
| FFG vs BMG | 0.67(-20,21) | 9.8(-10,31) | 4.6(-6.9,18) | 0.47 | 1 |
| VBG vs BMG | 0.065(-21,21) | 6.2(-12,26) | 2.8(-8,15) | 0.61 | 1 |
| VBG vs FFG | -0.19(-14,14) | -9.9(-36,15) | -1.8(-13,8.9) | 0.43 | 2 |
